# Perception of Smile Attractiveness in Excessive Gingival Display: A Cross-Generational Observational Study

**DOI:** 10.3390/dj14100661

**Published:** 2026-10-10

**Authors:** Ali Hassan Alfaifi, Damian J. J. Farnell, Jawaher Alalyah, Rahaf Alamri, Rahaf Alshehri

**Affiliations:** 1 College of Dentistry, King Khalid University, Abha 61421, Saudi Arabia; 2 School of Dentistry, Cardiff University, Cardiff CF24 0ZZ, UK

**Keywords:** excessive gingival display, smile attractiveness, gummy smile, perception

## Abstract

**Objectives**: Excessive gingival display (EGD), or “gummy smile,” is one of the most common esthetic issues among the young generation. However, the influence of age on the perception of gummy smile acceptance remains unclear. Hence, the current study aimed to evaluate whether smile attractiveness ratings for gingival display differ across generations and among observers. **Materials and methods**: A smiling photograph of a 24-year-old female was obtained and digitally modified in Photoshop to create six different images with gingival exposures. Participants aged 18–80 years evaluated the images, presented in randomized and repeated order, on an 11-point Likert scale (0 = not attractive; 10 = extremely attractive) and by sex, student status, and medical or dental affiliation using mixed-model repeated-measures ANOVA. Intra-rater reliability was assessed using test–retest, with intraclass correlation. **Results**: A total of 115 participants were included in the current cross-sectional study. Attractiveness varied significantly across the six different conditions (*p* < 0.05). The highest mean rating was obtained for 1 mm of tooth coverage (8.20 ± 1.48) and the lowest for 5 mm of gingival exposure (4.60 ± 2.25). Ratings did not differ significantly between age groups. Observer sex showed no significant main effect, although a small but significant sex × condition interaction was observed (*p* = 0.007). Student status and medical or dental affiliation showed no significant between-group main effects. Test–retest reliability was moderate to good (ICC 0.53–0.87; overall ICC = 0.82). **Conclusions**: Within the limitations of the current study, it could be concluded that perceived smile attractiveness is primarily influenced by the amount of gingival display. Neither age nor observer sex showed a significant main effect on attractiveness ratings, although the rating pattern across conditions varied slightly by sex. Gingival exposure of ≤3 mm was perceived as attractive, whereas 5 mm was consistently judged least attractive across all demographic subgroups.

## 1. Introduction

Smiling is the first human expression that reflects several emotions. It is the most important aesthetic feature and significantly influences the attractiveness and personality of an individual. Excessive gingival display (EGD), also known as “Gummy smile,” is defined as the exposure of more than 3 mm of maxillary gingiva during smiling [1]. Studies have reported that around 10% of individuals between the ages of 20 and 30 years suffer from EGD [2,3,4]. The prevalence of this condition is higher in females than in males [2,3,4]. 

The etiology of EGD is multifactorial, with anatomical and pathological variations. These include passive eruptions, short upper lips, hyperactive upper lips, vertically maxillary excess, and dental malocclusion [5]. These factors, independently or in combination, can lead to EGD. Additionally, EGD or skeletal origin is associated with the ‘long face syndrome’, which is characterized by the vertical growth of the maxilla leading to excessive dental and gingival display [6]. Although EGD is associated with facial appearance, it may result in reduced self-esteem, confidence, and, in some cases, depression. Al Jasser et al. reported that individuals with EGD face constant social and psychological challenges due to their smile [7]. 

Gingival display can influence smile attractiveness. Moreover, excessive gingival display requires therapeutic measures. The most acceptable treatment for EGD is lip repositioning and botulinum toxin A injections [4,8]. Another acceptable treatment is orthodontic treatment, which can provide an esthetic smile along with proper occlusal alignment [9]. However, the patient’s perception of the treatment outcome is of utmost importance, as the patient’s esthetic needs may differ from the clinical decisions made by dentists [10,11]. This perception may vary regarding patients’ age, gender, and connection to the dental field [11]. Hence, acknowledging the individual’s perception is required when assessing treatment need. Despite several published studies on EGD, there is still a gap in laypeople’s perceptions of individuals with EGD [12,13,14,15]. Moreover, there is always disagreement regarding EGD among laypeople and dentists. Studies have reported that laypeople are relatively insensitive to small degrees of gingival display, typically not perceiving it as unattractive until it exceeds approximately 3–4 mm [12,13,14,15], with attractiveness declining as exposure increases beyond this point. Even with the growing body of literature, data are limited on laypeople’s perception of EGD and the converse condition of increasing tooth coverage. Hence, the current cross-sectional study aimed to evaluate whether the perceived attractiveness of a smile with progressively altered gingival display differs across generations of laypeople and to explore whether sex, student status, and affiliation with the medical or dental field influence these attractiveness evaluations. The null hypothesis was that attractiveness ratings for smiles with excessive gingival display would not differ between participants aged ≥ 40 and <40 years.

## 2. Materials and Methods

### 2.1. Study Design and Ethical Approval

This cross-sectional, observational, cross-generational study was approved by the Dental School Research Ethics Committee at Cardiff University (Ref. No. 2319). A 24-year-old female volunteer agreed to participate in the study and provided written consent, along with a smile photograph, to be included in the evaluation. The study followed the revised Declaration of Helsinki guidelines. The objective of the study was explained to the participants, written informed consent was obtained, and the study was reported following STROBE recommendations. 

### 2.2. Participants and Recruitment

The participants were selected using convenience sampling. Participants aged 18–80 years were included, and they were divided into two groups: group 1 (18–40 years) and group 2 (40–80 years). Participants younger than 18 years, and those with any psychological anomalies were excluded from the study. The current study was conducted amongst the students and staff working in Cardiff University as the study was part of the master thesis. The data were collected in two ways: online (for participants who were unable to travel) and face-to-face. In the online survey, the link was shared with participants via a Cardiff University-accredited platform. Potential participants were approached, and informed consent was taken. After receiving informed consent, participants’ demographics (age, sex, occupation, and university course, wherever applicable). Participants were asked whether they were dentists or dental students to maintain strict inclusion criteria, as this may affect the study’s outcome. 

### 2.3. Image Preparation

**Figure 1:**
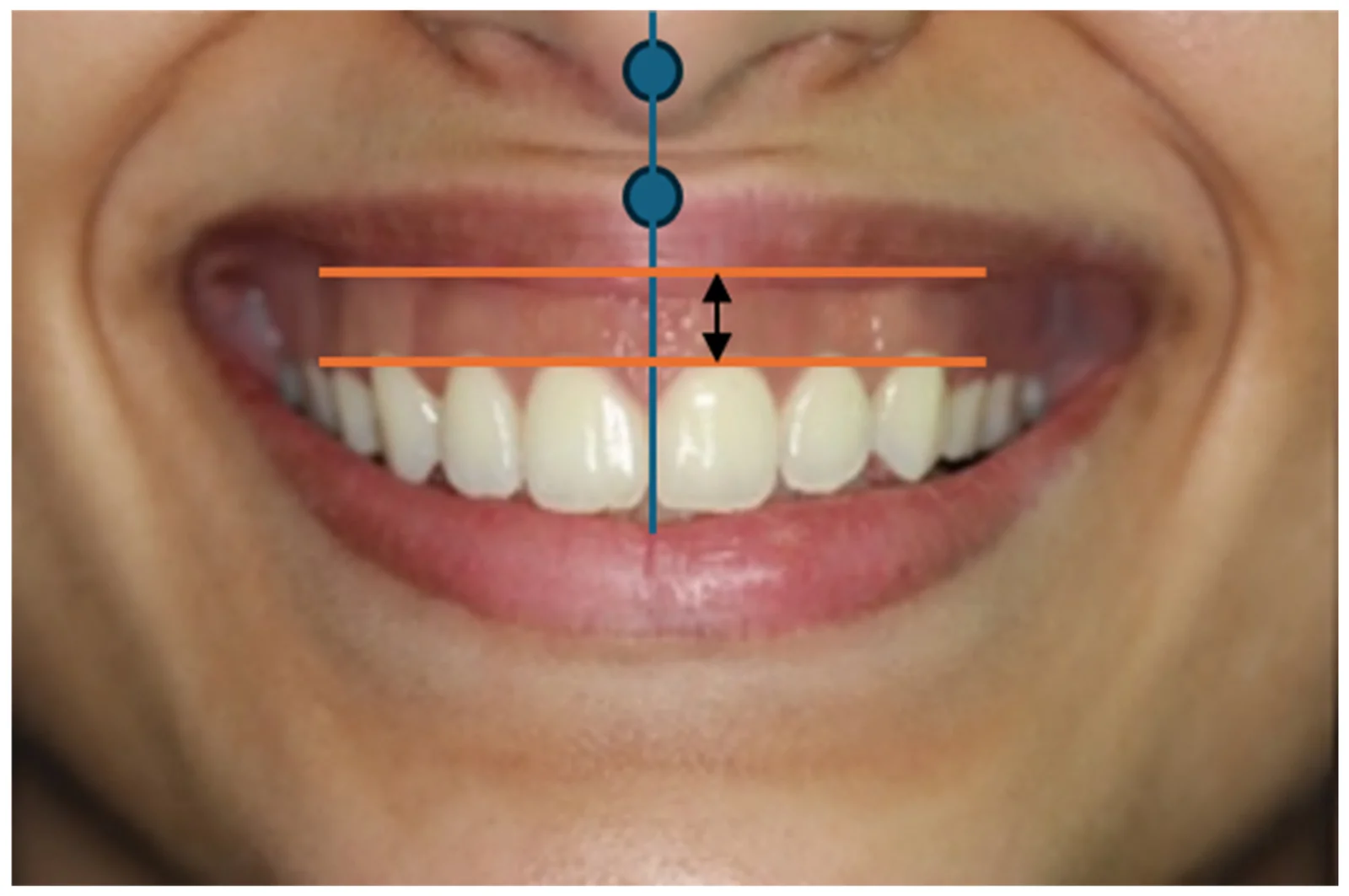
Illustration of the method used to create images of different gingival displays.

The images were generated according to the methods described in previously published studies (Figure 1) [11,15,16,17,18]. Two points were marked at the subnasal region, corresponding to the upper limits and lower limits of labial filters to the sagittal plane just above the vermilion border of the lips. Following this, two horizontal lines were drawn, one parallel to the uppermost gingival line and another parallel to the inferior border of the lips, both perpendicular to the vertical line. Finally, the images were created following the amount of gingival exposure. 

**Figure 2:**
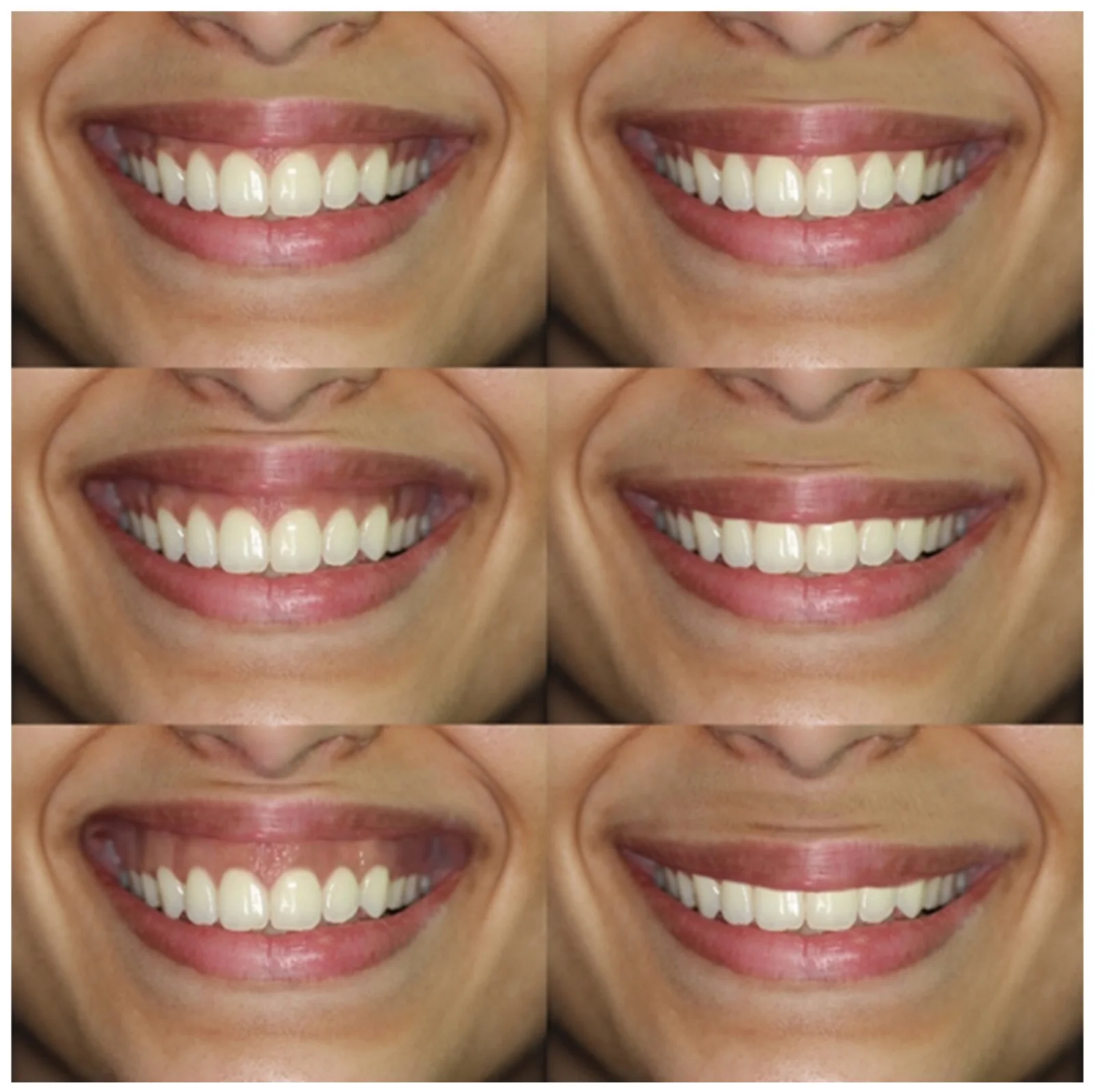
Edited images showing different levels of gingival exposure and teeth coverage. (Exposure 1) 1 mm of gingival exposure (**top left**). (Coverage 1) 1 mm of teeth coverage (**top right**). (Exposure 3) 3 mm of gingival exposure (**middle left**). (Coverage 3) 3 mm of teeth coverage (**middle right**). (Exposure 5) 5 mm of gingival exposure (**bottom left**). (Coverage 5) 5 mm of teeth coverage (**bottom right**).

The altered images were generated from a single standardized photograph. Photographs were captured with a digital single-lens reflex camera (Canon EOS Rebel) fitted with a 100 mm macro lens (EF 100 mm f/2.8L IS USM) and a ring flash (Canon Macro Ring Lite MR-14EX II). Using image-editing software (Adobe Photoshop v23.5.5), the upper lip was progressively repositioned relative to the gingival margin of the right maxillary central incisor (the reference point) to simulate 1, 3, and 5 mm of gingival exposure above the margin and 1, 3, and 5 mm of gingival coverage (Figure 2). 

### 2.4. Rating Procedure

The six images were included in the survey and presented in a randomized order. Each image was presented twice to each observer. The two ratings for each image were averaged to produce a single mean score per observer per image, which was used in the primary repeated-measures analysis. The two repeated ratings were also used to assess intra-rater reliability via the ICC. Raters scored the attractiveness of each smile on an 11-point Likert scale from 0 (not attractive) to 10 (extremely attractive). Responses were collected through a password-protected Google Form and exported to a password-protected spreadsheet for labeling and preliminary analysis.

### 2.5. Statistical Analysis

The sample size was calculated using the G*Power online sample size calculator with an alpha of 0.05 and a power of 85% (G*Power 3.1.9.7; Heinrich-Heine Universität Düsseldorf, Düsseldorf, Germany) to be at least 115 participants. After collecting the required scores, the data were stored in an Excel spreadsheet (Microsoft Corporation, Redmond, WA, USA). The Statistical Package for the Social Sciences (version 27.0; SPSS Inc., Chicago, IL, USA) was used to analyze the data. Descriptive statistics (mean, median, standard deviation, and confidence intervals) were calculated. A mixed-model repeated-measures ANOVA was conducted with gingival condition (six levels) as the within-subjects factor and observer group (age group, sex, and student status, analyzed in separate models) as the between-subjects factor. Each image was rated twice by every observer; the two ratings were averaged to yield a single score per condition, which was used in the primary analysis. Sphericity was assessed using Mauchly’s test, and the Greenhouse–Geisser correction was applied where sphericity was violated. Test-retest reliability was assessed using absolute-agreement intraclass correlation coefficients (ICC) and Spearman correlation coefficients. Significance was set at *p* < 0.05, with partial eta squared (η^2^_p_) as the effect size.

## 3. Results

### 3.1. Participant Characteristics

Of the 130 individuals approached, 10 did not respond and 5 returned incomplete surveys and were excluded, leaving 115 participants with complete responses for analysis (88.5%). Most participants were in between the age group of 18–40 years old (*n* = 69; 60.0%) and female (*n* = 82; 71.3%). Most of the participants were not students (n = 96; 81.7%) and reported no medical or dental background (*n* = 79; 68.7%); 47 participants (40.9%) reported some affiliation with or exposure to the dental field. Participant characteristics are summarized in Table 1. 

### 3.2. Attractiveness Across Levels of Gingival Display

**Figure 3:**
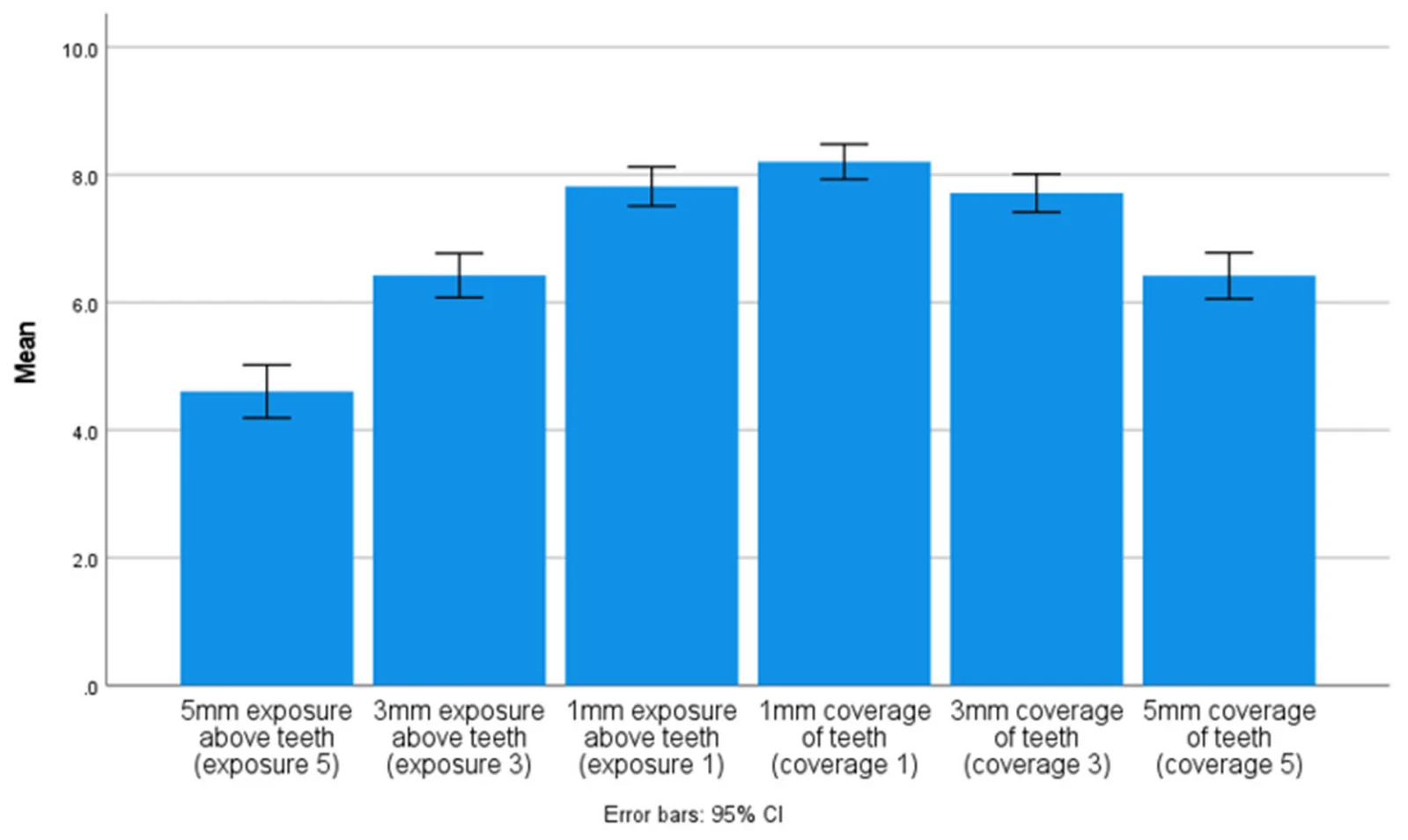
Mean attractiveness ratings (±95% confidence interval) for each level of gingival exposure and tooth coverage, ordered from maximal gingival exposure (left) to maximal tooth coverage (right). The whisker (error) line marks the midpoint of the scale (error bars representing the 95% confidence interval). Ratings peak at minimal alteration (1 mm coverage) and fall below the midpoint only at 5 mm of gingival exposure.

Mean attractiveness ratings differed significantly across the six images (*p* < 0.05; Table 2, Figure 3). The smile with 1 mm of tooth coverage received the highest mean rating (8.20 ± 1.48), followed closely by 1 mm of gingival exposure (7.82 ± 1.66) and 3 mm of tooth coverage (7.71 ± 1.62). Ratings declined as the alteration increased in either direction, reaching their lowest value at 5 mm of gingival exposure (4.60 ± 2.25). 

### 3.3. Effect of Observer Age

Comparison of the two age groups revealed only minor differences in mean ratings, including a slightly higher mean for 5 mm of gingival exposure among older raters (group 2: 4.64; group 1: 4.58) (Table 3). The ANOVA showed that the within-subjects effect of condition remained highly significant (*p* ≤ 0.05), whereas neither the between-subjects main effect of age nor the age × condition interaction was significant.

**Figure 4:**
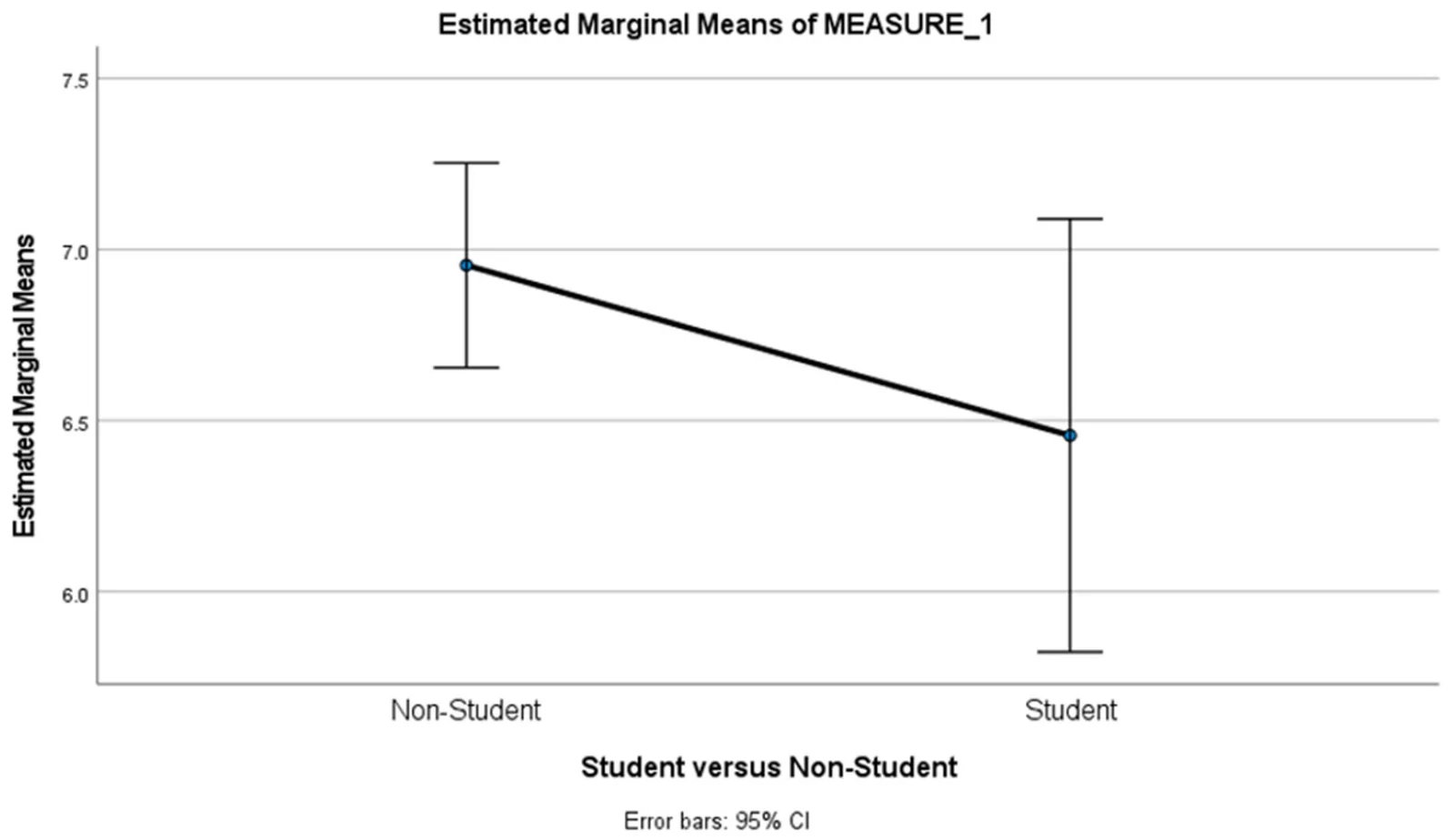
Estimated marginal means for student versus non-student observers across all levels of gingival exposure and coverage. Although students showed numerically lower mean ratings, this difference was not statistically significant (*p* = 0.051), and the 95% confidence intervals overlapped.

Mauchly’s test indicated a violation of sphericity (W = 0.195, χ^2^(14) = 183.12, *p* < 0.05); the Greenhouse–Geisser correction (ε = 0.62) was therefore applied. There was a significant main effect of condition, F (3.10, 350.24) = 146.40, *p* < 0.001, η^2^_p_ = 0.56, with ratings highest for 1 mm coverage (8.20) and lowest for 5 mm exposure (4.60). The main effect of age group was not significant, F (1, 113) = 0.002, *p* = 0.962, η^2^_p_ < 0.05, nor was the age × condition interaction, F (3.10, 350.24) = 0.98, *p* = 0.428, η^2^_p_ = 0.01. The main effect of sex was not significant, F (1, 113) = 0.13, *p* = 0.721, η^2^_p_ = 0.001; however, a small but significant sex × condition interaction was found, F (3.10, 350.24) = 3.24, *p* = 0.007, η^2^_p_ = 0.03. Neither the main effect of student status, F (1, 113) = 3.88, *p* = 0.051, η^2^_p_ = 0.03, nor the student × condition interaction, F (3.10, 350.24) = 0.80, *p* = 0.551, η^2^_p_ = 0.05, reached significance (Table 4 and Figure 4). 

Test-retest reliability was moderate to good, with single-measure ICCs ranging from 0.53 to 0.87 (overall ICC = 0.82, 95% CI 0.74–0.88) and Spearman coefficients from 0.59 to 0.87 (Table 5).

## 4. Discussion

This study examined perceptions of EGD for smile attractiveness among people of different age groups. The key finding is that perceived attractiveness was determined by the magnitude of gingival exposure or tooth coverage, and no significant difference was observed with the observer’s age, accepting the null hypothesis. Minimal gingival displays were rated favorably, with 1 mm of tooth coverage and 1 mm of gingival exposure receiving the highest scores, whereas 5 mm of gingival exposure was consistently judged as the least attractive. 

In the current study, the attractiveness of different levels of gingival display of upper incisors (ranging from 5 mm exposure to 5 mm coverage) on the photograph of a female smile was used to record the findings. The smile attractiveness was scored using a Likert scale. Alaqeely et al., in their study, compared smile attractiveness in photographs of males and females with gingival exposure ranging from 4mm to −4mm, respectively, and recorded smile attractiveness using visual analogue scale scores [19]. Studies have reported that Likert-scale evaluations can yield precise outcomes and are easy to follow, even for laypeople [2,19,20]. Hence, this study used Likert scoring to analyze smile attractiveness. Couper et al. found that, although visual analysis and scoring require time, they are better measurement techniques than numeric input or radio buttons [21]. Kokich et al. performed a study to quantify smile perception among dentists and laypeople using static photos [12]. They evaluated eight esthetic criteria ranging from crown length, crown width, incisor angulation, midline, open gingival embrasure, gingival margin, incisal plane, and gingiva-to-lip distance, to assess and compare smile attractiveness [12]. 

All the examiners gave higher scores on the images showing little gingival display (mean = 8.20; SD = 1.48) in the current study. Similarly, Alaqeely et al. reported that the most pleasing smile, as scored in the VAS system, was one with minimal gingival exposure [19]. Peck et al. stated that gingival exposure up to 1 mm is considered aesthetically pleasing [18]. This result also suggests that the most pleasing smiles are those in which no gingiva is exposed. In a systematic review, it was reported that laypeople and dentists have different perceptions of smile aesthetics, mainly because dentists’ perceptions may be influenced by the concepts they are taught in school [6]. In the current study, no significant difference was reported among participants influenced by dental connections. 

In the current study, no significant difference was observed in the outcome by age group. However, intergroup differences in scoring indicated that a similar aesthetic outlook was present amongst the group members. The results of the current study differ from those of other published studies, mainly due to methodological differences [8,19,22,23]. Kokich et al. conducted a similar study but changed the thickness and shape of the upper lips; hence, it was difficult for laypeople to differentiate [12]. Furthermore, beauty is neither purely subjective nor purely objective; alongside individual variation, it is shaped by quantifiable parameters such as proportion, symmetry, and harmony. Overall, smile perception showed no significant difference across age groups and instead varied according to the individual observer’s sensitivity.

Variations in the distance between the upper lip and the upper incisors have been created in the images of this study, including 5 mm gingival exposure, 3 mm gingival exposure, 1 mm gingival exposure, 1 mm tooth coverage, 3 mm tooth coverage, and 5 mm tooth coverage. In a cross-sectional study, orthodontists, lay people, and general dentists evaluated the images and considered gingival exposure of ≤4 mm acceptable [11]. Another study reported that participants with prior orthodontic treatment were more sensitive in scoring smile attractiveness and rated 0 (minimal gingival exposure) as a pleasing smile [10]. Studies published from Japan and Jordon reported that laypeople found smiles with gingival exposure of 2–3 mm attractive [24,25]. In the current study, participants in the United Kingdom found smiles with 1mm of gingival exposure to be attractive. Similar findings were reported in a study conducted in Saudi Arabia, where participants found gingival exposure of less than 1mm attractive [19]. This disagreement could be attributed to differences in culture and environmental factors. 

A key constraint of this study is that all six conditions were derived from a single model. This design maximized internal validity by holding facial morphology, sex, age, and ethnicity constant, thereby ensuring that variation in ratings reflected the manipulated smile parameters rather than differences between faces. It does, however, limit the extent to which the findings can be generalized. Perception of smile aesthetics is known to be influenced by the characteristics of the face being judged; the same degree of gingival display may be evaluated differently on a male versus a female face, on younger versus older faces, and across differing ethnic backgrounds and facial proportions, since aesthetic norms and the perceived acceptability of gingival exposure are not uniform across these groups. The specific attractiveness thresholds reported here should therefore be understood as applying to a face of this profile, and the exact tipping points may shift for faces differing in sex, age, ethnicity, or morphology. The broad pattern observed is consistent with prior literature [19,24,25], which suggests the general direction of the effect may hold more widely; however, confirming this, and establishing whether the quantitative thresholds are stable across face types, would require studies using multiple models spanning a range of sexes, ages, and ethnic backgrounds. 

Several limitations should be acknowledged. The number of participants varied across subgroups, and the large sample included younger and female participants, limiting the precision of subgroup comparison. Only a single female volunteer has provided her image, limiting the generalizability of facial types, sexes, and ethnic backgrounds. Additionally, the standardized study design was not designed for reproducibility between studies. Repeated presentation of each image, however, allowed for the estimation of intra-rater agreement. The participant characteristics examined here (student status and medical/dental affiliation) serve as representations and do not directly capture education level or socioeconomic status. The sample was imbalanced in sex (71.3% female), age (60% under 40), and occupation (81.7% non-students); as women and younger individuals have been reported to be more critical of smile aesthetics and gingival display, this may have influenced the ratings. Finally, data were collected through both online and face-to-face modes, and display parameters (screen type, size, brightness, resolution, and contrast) could not be standardized in the online arm; as the face-to-face sample was comparatively small, a formal subgroup comparison between modes was not feasible, and the potential influence of unstandardized displays on ratings cannot be entirely excluded.

## 5. Conclusions

Within the limits of this study, smiles with minimal gingival display (1 mm exposure and 1 mm coverage) received the highest attractiveness ratings. This pattern was consistent across age groups, and age, student status, and dental-related background were not significant determinants of perceived attractiveness; a small but significant sex × condition interaction was observed. Because all ratings were based on a single 24-year-old female face and participants were recruited at one institution, these findings should be interpreted as applying to this specific facial profile and sample rather than generalized across sexes, ages, ethnic groups, or populations. Within these constraints, the results underscore the value of considering individual patient characteristics and the perceptions of their social audience, rather than applying uniform esthetic criteria, when planning the management of excessive gingival display.

## Figures and Tables

**Table 1:** Demographic characteristics of the participants.

Characteristic	Category	n	%
Age group	18–40 years	69	60.0
	40–80 years	46	40.0
Sex	Female	82	71.3
	Male	33	28.7
Student status	Non-student	96	81.7
	Student	19	18.3
Medical/dental background	No	79	68.7
	Yes	36	31.3
Dental exposure	No	68	59.1
	Yes	47	40.9

n = sample size; % = percentage.

**Table 2:** Descriptive statistics for attractiveness ratings (0–10 scale); n = 115 for all conditions, with no missing data.

Condition	Mean	Median	SD	Min	Max
5 mm gingival exposure	4.60	4.50	2.25	0.5	10.0
3 mm gingival exposure	6.42	6.50	1.87	0.5	10.0
1 mm gingival exposure	7.81	8.0	1.66	2.0	10.0
1 mm tooth coverage	8.20	8.50	1.48	3.0	10.0
3 mm tooth coverage	7.71	8.00	1.61	3.5	10.0
5 mm tooth coverage	6.41	6.50	1.95	1.0	10.0

SD = standard deviation.

**Table 3:** Mean (SD) attractiveness ratings by age group.

Condition	18–40 Years (n = 69)	40–80 Years (n = 46)
5 mm gingival exposure	4.58 (2.36)	4.64 (2.09)
3 mm gingival exposure	6.47 (1.96)	6.33 (1.74)
1 mm gingival exposure	7.80 (1.72)	7.83 (1.58)
1 mm tooth coverage	8.32 (1.46)	8.02 (1.50)
3 mm tooth coverage	7.74 (1.69)	7.66 (1.51)
5 mm tooth coverage	6.27 (2.09)	6.63 (1.72)

**Table 4:** Mixed-model repeated-measures ANOVA of smile attractiveness ratings.

Source	F	df	*p*	η^2^_p_	Sphericity Correction
Within-subjects effect					
Condition (gingival level)	146.40	3.10, 350.24	<0.05	0.564	Greenhouse–Geisser
Model 1: Between-subjects factor: Age group (18–40 years and 40–80 years)					
Age group	0.00	1, 113	0.962	<0.05	—
Age group × condition	0.98	3.10, 350.24	0.428	0.009	Greenhouse–Geisser
Model 2: Between-subjects factor: Sex (Female vs. Male)					
Sex	0.13	1, 113	0.721	0.05	—
Sex × condition	3.24	3.10, 350.24	0.007	0.028	Greenhouse–Geisser
Model 3: Between-subjects factor: Student status					
Student status	3.88	1, 113	0.051	0.033	—
Student status × condition	0.80	3.10, 350.24	0.551	0.05	Greenhouse–Geisser

Three separate mixed-model repeated-measures ANOVAs were run, each with gingival condition (six levels) as the within-subjects factor and one grouping variable as the between-subjects factor; the within-subjects main effect of condition was equivalent across models and is reported once. Mauchly’s test indicated that sphericity was violated (W = 0.195, χ^2^(14) = 183.12, *p* < 0.001); the Greenhouse–Geisser correction (ε = 0.62) was applied to all within-subjects effects, and corrected degrees of freedom are reported. η^2^_p_ = partial eta squared. Group sizes: age 18–40 years = 69, 40–80 years = 46; female = 82, male = 33; non-student = 96, student = 19.

**Table 5:** Test–retest reliability of smile attractiveness ratings by condition.

Condition	ICC (95% CI)	Spearman ρ (95% CI)	*p*
5 mm exposure	0.865 (0.810–0.900)	0.869 (0.820–0.910)	<0.05
3 mm exposure	0.690 (0.550–0.790)	0.644 (0.520–0.740)	<0.05
1 mm exposure	0.545 (0.260–0.720)	0.618 (0.490–0.720)	<0.05
1 mm coverage	0.731 (0.630–0.810)	0.724 (0.620–0.800)	<0.05
3 mm coverage	0.531 (0.380–0.650)	0.586 (0.450–0.700)	<0.05
5 mm coverage	0.809 (0.730–0.860)	0.814 (0.740–0.870)	<0.05
Overall (6-condition scale)	0.824 (0.740–0.880)	0.812 (0.740–0.870)	<0.05

ICC = intraclass correlation coefficient, two-way random-effects model, absolute agreement, and single-measure [ICC]. All coefficients were statistically significant (*p* < 0.001). The overall row reflects the mean rating across all six conditions (test vs. retest).

## Data Availability

The datasets generated and analyzed during the study are available from the corresponding author on reasonable request.
